# Nurses respond to patients’ psychosocial needs by dealing, ducking, diverting and deferring: an observational study of a hospice ward

**DOI:** 10.1186/s12912-015-0112-8

**Published:** 2015-11-17

**Authors:** Hazel Hill, Josie MM. Evans, Liz Forbat

**Affiliations:** School of Health Sciences, University of Stirling, Stirling, FK9 4LA UK; Australian Catholic University and Calvary Health Care, Canberra, 2600 Australia

**Keywords:** Hospice nursing, Psychosocial, Observation, Palliative

## Abstract

**Background:**

Psychosocial support is considered a central component of nursing care but it remains unclear as to exactly how this is implemented in practice. The aim of this study was to provide a descriptive exploration of how psychosocial needs (PNs) of patients in a hospice ward are expressed and met, in order to develop an understanding of the provision of psychosocial support in practice.

**Methods:**

An embedded mixed-methods study was conducted in one hospice ward. Data collection included observations of patients’ expressions of PNs and nurses’ responses to those expressed PNs, shift hand-overs and multi-disciplinary meetings. Interviews about the observed care were conducted with the patients and nurses and nursing documentation pertaining to psychosocial care was collated. Descriptive statistical techniques were applied to quantitative data in order to explore and support the qualitative observational, interview and documentary data.

**Results:**

During the 8-month period of observation, 227 encounters within 38 episodes of care were observed among 38 nurses and 47 patients. Within these encounters, 330 PNs were expressed. Nurses were observed immediately responding to expressed PNs in one of four ways: dealing (44.2 %), deferring (14.8 %), diverting (10.3 %) and ducking (30.7 %). However, it is rare that one type of PN was clearly expressed on its own: many were expressed at the same time and usually while the patient was interacting with the nurse for another reason, thus making the provision of psychosocial support challenging. The nurses’ response patterns varied little according to type of need.

**Conclusions:**

The provision of psychosocial support is very complex and PNs are not always easily recognised. This study has allowed an exploration of the actual PNs of patients in a hospice setting, the way in which they were expressed, and how nurses responded to them. The nurses faced the challenge of responding to PNs whilst carrying out the other duties of their shift, and the fact that nurses can provide psychosocial support as an inherent component of practice was verified. The data included in this paper, and the discussions around the observed care, provides nurses everywhere with an example against which to compare their own practice.

## Background

Psychosocial care is a component of all nurses’ work [1, 2]. Palliative care, in particular, has psychosocial care as an essential focus [3–5]. Palliative care patients’ psychosocial needs (PNs) have been identified in research studies in a number of ways. Some studies have identified PNs by enquiring directly about them [6, 7]. Other researchers have inferred the presence of PNs by discussing patients’ and/or nurses’ views about their experiences of care [8–10], with studies that assess satisfaction or quality of care assessment arguably also falling within the psychosocial sphere [11, 12].

A wide variety of PNs are reported in a variety of ways in existing literature, for this study these were categorised by the researchers into four groups: rights, coping, identity, and expression.

‘Rights’: Patients’ need for self-determination, safety, and security. These include the wish to be self-determining [13, 14], through continued involvement in decision-making [8, 15] and autonomy, to the level patients desire [16, 17]. Alongside these are the rights to maintaining maximum quality of life [6, 18] and independence [9, 19], being treated with dignity [19, 20], given privacy [14, 21] and feeling safe and secure [22, 23].

‘Coping’: The need for patients to have understanding and acceptance of their condition and their approaching death, whilst maintaining hope. Palliative patients need to adjust and cope [6, 8] with many changes occurring as a consequence of deterioration in their condition which can be facilitated through understanding [24, 25] and acceptance [24, 26]. Coping includes PNs around fear for the future [27] and of death [28].

‘Identity’: The need for patients to have feelings of self-worth and to sustain relationships where possible. Patients seek to maintain an identity [17, 26] as an individual [8, 29] with a continuing role in life [24, 30] rather than assuming the persona of ‘patient’. Sustaining relationships [22, 26] and creating companionships [31, 32], through another PN: communication [23, 33], assists patients to meet PNs surrounding having a positive self-concept [17, 34] and self-esteem [30, 35].

‘Expression’: The feelings palliative care patients have and how they express them [36–38], ranging from elation to despair and the desire for quality of life. These include anxiety and depression.

Despite numerous studies identifying the PNs expressed by palliative care patients, there is very little empirical evidence on how nurses actually provide psychosocial care in practice alongside their other duties in busy ward environments [19, 39, 40]. It may be that as psychosocial care is recognised as a fundamental aspect of palliative care for all practitioners [41–43], nurses leave this aspect of care to their colleagues from other disciplines. The aim of this study was to investigate the types of PNs expressed by patients in a palliative care setting and how nurses immediately responded to them; in other words how nurses operationalise the term psychosocial support.

## Methods

This study used an embedded mixed-methods approach [44] to explore patients’ expressions of PNs and nurses’ responses to them in a hospice ward in Scotland, which serves both urban and rural populations. Participant observation was combined with qualitative interviews and analysis of nursing documentation, such as care records.

The study site was a 24 bedded ward, with both single and shared rooms, in a specialist palliative care unit. Patients had active, progressive, non-curative diseases (90 % had a malignancy; the majority of the remaining patients had a neurological illness). Patients were admitted to the hospice with at least one of the following five care aims: symptom management, therapeutic respite, terminal care, assessment or rehabilitation. The researcher (Hazel), an experienced palliative care nurse, completed this study, whilst employed as a research nurse practitioner, in order to gain a PhD. Having previously known the researcher as an educator in palliative care, the nurses were aware of her background and contributed to discussions around the study’s aims design. The researcher worked on the ward during the study design period to become an unobtrusive member of the ward team to minimise researcher impact [45], then adopted a participant-as-observer role [46]. A reflexive diary was kept throughout the duration of the study to identify and balance researcher bias.

Information sheets were distributed to all registered (RGN) and auxiliary (AuxN) nurses working day-duty on the ward and 38 (88 %) gave written consent for their care to be observed. Twenty-three were RGNs, fifteen were AuxNs. The five nurses who did not offer to participate were all AuxNs. The researcher then introduced herself to all ward patients. Patients who were cognitively intact, and not thought to be in the last few days of life, were offered an information sheet outlining the research. After at least 24 hours, during which time patients were encouraged to discuss the study with their significant others, they were approached for written consent. 47 patients (67.5 % of those eligible) provided consent, which was re-checked verbally throughout the duration of the study; 12 patients approached declined to participate. Participant characteristics are shown in Table 1.

**Table 1:** Participant characteristics

Patient characteristics (*n = 47*):		Nurse characteristics (*n = 38*):	
Age	Range: 38-91 years	Age	Range: 22-59 years
	Mean: 65.1 years		Mean: 44.47 years
Sex	Male: 19 (40.4 %)	Sex	Male: 0 (0 %)
	Female: 28 (59.6 %)		Female: 38 (100 %)
Average days spent in hospice at time of observation	Range: 1-221 days	Role	Registered General Nurse: 23 (60.5 %)
	Mean: 31.7 days		Auxiliary Nurse: 15 (39.5 %)
Care aim	Assessment: 5 (10.6 %)	Education in psychosocial care	None: 5 (13.2 %)
	Rehabilitation: 2 (4.3 %)		Study day: 5 (13.2 %)
	Respite: 9 (19.1 %)		Short course: 11 (28.9 %)
	Symptom Control: 20 (42.6 %)		Module: 17 (44.7 %)
	Terminal Care: 11 (23.4 %)		
Diagnosis	Cancer: 39 (83 %)	Years of palliative care experience	Range: 0.5-19 years
	Neurological: 8 (17 %)		Mean: 8.7

On each shift, a nurse was selected to be observed, depending upon her availability over the subsequent days for interview. Sampling matrices were used to ensure greatest possible variety of nurse roles and times throughout ward day duty. Patient sampling occurred by chance by being the first consenting patient to express a PN to the observed nurse that shift. This approach resulted in a large convenience sample with a high degree of variety [45, 47].

### Data collection

Observation took place over an eight month period. The observational skills the researcher had developed during her nursing career were enhanced through extensive reading on observation as a research tool and discussions with her PhD supervisors. Wearing a different uniform to distinguish herself from the other nurses, the researcher worked alongside consenting nurses. If a consenting patient expressed a PN, as defined by Thomas et al. (2001), data collection commenced. A description of the observed care was captured by digital audio-recording notes immediately after the observation. This was transcribed as soon after the interaction as possible and shared with participants to verify the account. Semi-structured interview schedules, for both patient and nurse, were then created around the PNs, the nurses’ responses to them, and other emerging issues [48, 49]. Participants were only interviewed once. Nurse documentation and discussions around the observed PNs were also recorded, including shift handovers and multidisciplinary meetings.

Data collection ceased when a substantial sample size [50] produced clear, supportable claims.

### Analysis

Data was analysed primarily by the researcher and verified independently by two experienced researchers. The first seven interactions formed a pilot study. Among the consenting patients and nurses, an episode of care was defined as a discrete period of time during which a nurse worked with a patient to provide a specific aspect of care. Within these episodes of care, an encounter was defined as one nurse’s response to one or more PNs expressed by a patient at one time. These were categorised into PNs relating to rights, identity, coping, and expression.

All qualitative data relating to encounters were entered into an NVivo electronic qualitative analysis software project and descriptive analysis [49] began during transcription of the first observation. This analysis identified key concepts which emerged from the data and were compared to each subsequent nurse-patient interaction to generate propositions.

During analysis a categorisation of nurse responses emerged, entitled the ‘4Ds’. Chi squared tests were carried out to determine whether type of PN (rights, identity, coping, and expression) was related to the nurses’ responses (‘dealing’, ‘deferring’, ‘ducking’, or ‘diverting’).

Findings were verified with participants during subsequent data collection and discussed with nurse participants via presentations.

### Ethical approval and consent

Ethical approval was given by Forth Valley Research Ethics Committee (04/S0604/14) and NHS Research and Development Office. Confidentiality was maintained by the use of pseudonyms and ensuring any potentially identifying details were removed from all data.

## Results and discussion

227 encounters within 38 episodes of care were observed among 38 nurses and 47 patients. Within these encounters, 330 PNs were expressed. All of the PNs outlined in the literature summarised above were expressed at some point during the fieldwork in the hospice. A maximum of eight were expressed during any one encounter.

Analysis of the observational data identified that nurses immediately responded to patients’ PNs in one of four ways: ‘dealing’, ‘deferring’, ‘ducking’, or ‘diverting’. Nurses could acknowledge the PNs and ‘deal’ with it directly in accordance with the patient’s wishes. Some nurses recognised that a PN had been expressed but ‘deferred’ dealing with it, either until later or until another hospice healthcare professional (HCP) could deal with it. At times nurses would realise that a patient had a PN but would ‘divert’ their support to another aspect of care that would benefit the patient. Alternatively, nurses did not acknowledge the patient’s signal at all, effectively ‘ducking’ the PN as if it had not been expressed. The nurses were observed using different responses during most episodes of care, ranging from one type of response to all four. However, these labels do not imply any judgment as to the appropriateness of the nurses’ actions; for each type of response there may be valid reasons for that particular response.

Table 2 shows the distribution of responses within each category of type of PN. Overall, the nurses ‘dealt’ with around 44 % of needs, and ‘ducked’ around 30 %, while ‘deferring’ and ‘diverting’ rates were around 15 % and 10 % respectively.

**Table 2:** Distribution of response type against category of psychosocial need

Response	Type of psychosocial need expressed				Total per response
	Rights	Identity	Coping	Expression	
Ducking	45 (27.3 %)	18 (36 %)	13 (31 %)	25 (34.2 %)	101 (30.6 %)
Deferring	25 (15.2 %)	1 (2 %)	8 (19 %)	15 (20.5 %)	49 (14.8 %)
Diverting	20 (12.1 %)	2 (4 %)	3 (7.1 %)	9 (12.3 %)	34 (10.3 %)
Dealing	75 (45.5 %)	29 (58 %)	18 (42.9 %)	24 (32.9 %)	146 (44.2 %)
Total	165	50	42	73	330

The patterns of responses to PNs relating to rights and coping displayed similar proportions to the overall sample. When identity PNs were expressed, compared to the three other types, nurses tended to either ‘deal’ with them or not: ‘deferring’ or ‘diverting’ was the response for only three of these PNs (χ^2^ = 11.57, p < 0.01). There was also a statistically significant lower proportion of expression PNs that were immediately ‘dealt’ with (as opposed to ‘ducked’, ‘deferred’ or ‘diverted’) compared with the other types of PNs (χ^2^ = 6.18, p = 0.01). These findings suggest that there may be some association between the type of PN expressed and the response provided.

### Dealing

When encounters were assigned to the ‘dealing’ category the nurse was either observed dealing with a patient’s PN, or had described the provision of psychosocial support in documentation or liaison. 104 PNs were ‘dealt’ with. Allocating encounters to the dealing group was, in the majority of cases, straightforward: a PN was expressed and immediately dealt with. However, nurses also demonstrated ‘dealing’ when patients did not explicitly express a PN; this occurred in three ways: (i) recognising implied PNs, (ii) adapting nursing practice, and (iii) responding to previously expressed needs.

Detecting implied PNs are exemplified in the following excerpt where a patient, Wendy, was to attend the local hospital for an x-ray and requested to spend some time at the shops after her appointment. This was the first time she had tried shopping since her condition had deteriorated. As Ellen (RGN) and Hazel were helping Wendy to get ready for her trip out of the hospice, she started to talk about going to the shops:

### Fieldnotes

*Wendy was talking excitedly about going to the shops after her x-ray, ‘but I’m not sure how long I'll be, I do get very weak all of a sudden and if that happens I’ll just need to come back’. She appeared despondent about this. Ellen suggested ‘why don’t you take a wheelchair with you? You don’t have to use it, but it would be there as a safety-net and if you do get too weak your husband can push you round in it. That way you won’t have to come home until you are ready.’ Wendy was quiet, then after a short while replied ‘hmm, I’m not keen on taking a wheelchair’. Ellen said ‘okay, but if you change your mind before you go, just say.’*

### Patient interview

*Hazel: “Sometimes [*nurses*] persuade you to do things, such as when you went out the other day taking the wheelchair with you. Did you feel okay about us doing that to you?”*

*Wendy: “Yes, I did. I wouldn’t have asked for a wheelchair, but I was glad of the opportunity of having one, knowing that for several months previously I would have died to have had a wheelchair to sit in…it was quite good to know that I had the opportunity to use it, I didn’t need it, but the opportunity was there for me.”*

Ellen’s suggestion to use the wheelchair, and the way it was put to Wendy, had positive impacts on Wendy’s psychosocial well-being. Wendy had control over whether to take the chair; taking the chair gave her a sense of security; the time she had at the shops gave her a chance to be herself, doing something she enjoyed, and the time with her husband allowed them to have a ‘normal’ interaction. Thus Ellen’s intuitive actions dealt with Wendy’s PNs.

‘Dealing’ encounters also occurred when psychosocial support was provided by the nurses adapting their behaviour, and/or actions, to provide care in a way that was preferred by, but not essential for, a patient. Care would still be effective without this change of practice, but by the nurse adapting their style of care, a patient could meet a number of PNs.

Other ways in which nurses changed their behaviour to interact with individual patients in order to offer psychosocial support related to the transfer of information. Some patients liked to be told about everything the nurse was doing for them, whereas other patients preferred the nurse to ‘*just do things’*. Some patients expected the nurses to know how to work with them and what their needs were, whereas other patients preferred to tell nurses about their condition. When nurses matched these patients’ preferences, encounters were classified as ‘dealing’, as the nurses were respecting the patient’s rights.

Thirdly, ‘dealing’ could relate to a previously expressed PN which had not yet been addressed. An example of this occurred when Ann (RGN) eventually found out that Bruce did not want to move to a single-room. During Bruce’s stay he had seen many other patients admitted to the bay and some had died. Several of the ward staff were concerned that witnessing these deaths was having a negative effect on Bruce, thereby warranting a move to a single-room.

### Fieldnotes

*Ann - who had hinted to Bruce this morning about moving to the single-room - said ‘I'll talk to Bruce about it’.*

*Ann told Bruce ‘there’s still another side-room available, but it’s up to you’. Bruce was not sure whether to go, saying ‘I would quite like to be able to play my music when I like without having to worry about other people, but I quite enjoy the company’. He seemed very hesitant to move to the single-room. After a short pause Ann suggested to him ‘but you're quite happy here, aren't you?’ and he said ‘yeah, so I'll stay here, today.’*

*After this conversation Ann told me ‘it was important that Bruce had the opportunity to make that choice’.*

Ann’s consideration of Bruce’s moving to a single room identifies a number of potential PNs, including: fear of dying, loss of relationships, anxiety, and the need for safety. These PNs were not discussed with Bruce during any observations. However, this example does demonstrate the nurse dealing with a PN that had previously been deferred by both her and others: giving Bruce the choice of whether to move rooms. The nurse put aside what she, and other members of the hospice staff, felt would be best for Bruce. The nurse focussed on what the patient wanted, thereby meeting a number of PNs, including autonomy and a sense of belonging.

The common factor in all of the ‘dealing’ encounters is that the nurse immediately supported the patients’ PNs.

### Deferring

Responses that involved ‘deferring’ occurred when nurses delayed dealing with a PN so that it could be dealt with at a later time, either by themselves or someone else. Forty-nine PNs were deferred, (some of which may have been responded to by ‘deferring’ initially and ‘dealing’ later). For a PN to be ‘deferred’ the nurse had to indicate to the patient that they had recognised the PN and that it would be dealt with later. This happened when Bruce’s need for information about his disease progression was ‘deferred’ to a later date by Evie (RGN).

### Fieldnotes

*Bruce said ‘there is one thing nobody's ever told me: what the results of those x-rays were that I had four weeks ago’. Evie paused for a wee while, then replied ‘oh, that's right, we must chase that up. Try not to worry about that just now.’*

Evie’s response to Bruce’s desire for information was representative of most of the ‘deferring’ responses, she indicated that she heard Bruce’s PN and attempted to placate him. Placation was a common response when nurses felt they required more information before a patient’s PN could be dealt with. What classifies Evie’s response as a ‘deferring’, rather than ‘ducking’, response is that immediately following Bruce’s episode of care, she reported his concern to a doctor.

‘Deferring’ encounters left the nurse with two options. In some cases, they would get another member of staff to deal with the PN because they felt that the other HCP had better skills or knowledge to deal with that situation. The alternative was that they would return to the patient themselves at a later time to offer support.

PNs were also ‘deferred’ when another HCP was directly involved in the episode of care. When other HCPs were working with a patient alongside a ward nurse, it was observed that the nurse always gave the HCPs control over what care should be provided. If a patient expressed a PN, and the other HCP did not pick up on this, the nurse was inhibited from dealing with the patient’s requirement but could return to deal with it later:

### Fieldnotes

*Later that morning, Marianne (RGN) was crouching beside Eliza’s bed obviously in deep conversation. When they had finished the conversation I asked Marianne about it. She told me, she had ‘gone back to discuss Eliza’s earlier concerns about her deteriorating condition. I didn’t deal with at the time because [the other HCP] had different things to discuss’.*

Nurses ‘deferred’ psychosocial support either because they felt they did not know enough about the patient and/or their PN or because they felt it was another staff member’s role to deal with the need. At other times, ‘deferring’ occurred when another member of staff redirected the conversation. However, when ‘deferring’ occurred nurses always showed patients they had recognised their PN and indicated that the required psychosocial support would be offered later.

### Diverting

When nurses used a ‘diverting’ response, the support they offered did not correspond with meeting the expressed PN: the nurses’ actions were aimed at meeting another need, which was not necessarily psychosocial. There were 34 PNs that were ‘diverted’. Nurses adopted a range of ways of ‘diverting’ PNs, for example, focussing on only one of a number of needs; offering practical solutions; and acting upon different care aims.

The first way nurses ‘diverted’ was by dealing with only part of a patient’s requirements, rather than addressing the patient’s full range of needs. It was common, in these circumstances, for a nurse to focus on patients’ physical needs and, often unwittingly, omit PNs. This type of ‘diverting’ occurred when Millie (RGN) was bed-bathing Flora:

### Fieldnotes

*A short time later Flora said ‘it’s about time I’m not here anymore’. Millie did not say anything for a while, then responded ‘things are much worse for you now?’ Flora agreed. Millie explained to Flora how her symptoms could be managed as her condition deteriorates, telling her ‘we’ll be able to keep you comfortable right up until the end’.*

In this example, Flora was expressing a number of PNs including worries about the future and difficulties coping with her deteriorating condition. Millie diverted the conversation away from these needs, rather than checking with Flora what her concerns were and allowing Flora to prioritise which to support.

The second type of ‘diversion’ was to offer an easily achievable practical solution to one issue, rather than exploring and managing the more complex but actual PN. For example, one patient Eliza liked to keep busy. Throughout her stay in the hospice she was always finding different ways to occupy her time. As her condition deteriorated, she continued to express a desire to find ways of occupying her time. However, instead of doing this, Marguerite (RGN) offered what she thought would be a quick solution to Eliza’s problem and Lily (RGN), facilitated this offer:

### Documentation

*“*[Eliza’s] *fed up with 4 walls, missing getting out of the room,* [query] *consider change of environment, move to* [another room] *would mean she could have patio doors open.” Marguerite.*

*“*[Eliza] *agreed to move to* [the other room] *and very pleased with brightness and open aspect.” Lily.*

In these excerpts of documentation both RGNs recognised that Eliza was unhappy with her current situation. However, their solution to this problem only had a temporary effect: Eliza’s boredom returned later that day and the move of rooms did not help her to accept her changing condition.

Differences in care priorities arose when patient expectations did not match the care offered. This occurred when Stuart, a patient whose mobility was deteriorating, wished to focus on improving his current mobility. The nurses’ aim was to support him to mobilise when he got home, with a consensus that Stuart’s mobility would not improve and, at best, he would be reliant on a wheelchair. Stuart had not come to terms with the fact that he would not regain full independence with his mobility. In an attempt to facilitate Stuart’s acceptance, the nurses had asked another HCP, who would also be involved with Stuart’s care on discharge, to come and talk to him about his mobility:

### Fieldnotes

*The HCP came in to talk to Stuart, as requested, when Camille (RGN), and I were bed-bathing him.*

*When Stuart, the HCP, and Camille were talking, Stuart mentioned ‘when I’m up walking’. Camille and the HCP looked at each other, then steered the conversation to talking about how Stuart would manage at home. Stuart said ‘but that’s in the future and I’m not ready to talk about that yet’.*

Although the nurses and Stuart were concerned about his mobility, their different care aims, and time, were preventing them from supporting Stuart to accept his changing condition. This incongruence between short and long-term goals of care meant that Stuart’s current PN was being diverted.

During all of the ‘diverting’ encounters the nurses responded to a patient’s needs. However, the support they provided did not deal with the patient’s immediate PNs.

### Ducking

‘Ducking’ responses were when a patient had a PN which the nurse did not attempt to support. In these circumstances no recognition was made by the nurse of the existence of the patient’s PN at the time it was expressed. There were 74 observed PNs that were ‘ducked’. ‘Ducking’ occurred under five conditions: (i) when nurses did not recognise PNs had been expressed; (ii) when the nurses’ current state of mind clouded their ability to respond; (iii) when nurses failed to engage with patients; (iv) when nurses did not want to disrupt the shift’s planned work; or (v) when the nurses felt not responding to the PN was in the patient’s best interest.

There were times when nurses simply did not recognise patients were expressing PNs. This most commonly occurred when patients hinted concerns about their disease progression:

### Fieldnotes

*After Nina (AuxN) and I finished assisting Eve to wash and dress, Nina supported Eve whilst she transferred into the arm-chair. Eve found this transfer difficult and had to rest during it. Both Eve and Nina’s moods were light-hearted and jovial throughout Eve’s care, even during the difficult transfer. However, when she was settled into the chair Eve’s mood changed and she sombrely said ‘you know, I was up and walking when I first came in here and now I can’t.’ Nina made no response to this.*

At interview, Nina told me she had not realised Eve was voicing worries about her condition. Nina related her inability to recognise Eve’s PNs to her lack of education concerning what PN are. Nina felt she had “*never had any training in psychosocial care*”.

For the remaining four groups of ‘ducking’ responses, the nurses were aware that PNs had been expressed, but did not respond. For example, on one occasion they stated that they ‘*had noticed a patient’s PN but chose not to respond*’. In one instance, a nurse who possessed the knowledge and skills to carry out psychosocial support and was observed on other occasions dealing with some very complex PNs, reported that she can temporarily lose her ability to respond to PNs:

### Nurse interview

*Annie: “there* [have] *been times when people have given me cues and I’ve been aware that I’ve not picked up on* [them]*, maybe because of the way I’ve been feeling at the time myself”*

Thirdly, nurses ‘ducked’ when they failed to engage with patients on a personal level when they were providing their care. This occurred when nurses were focused on the tasks of care rather than the requirements of the individual patient, or because the patient’s PN clashed with the only way the nurse could see of carrying out their duties. The latter is exemplified below as Sybil (AuxN) and Hazel transferred Polly to and from her chair.

### Nurse interview

*Hazel: “With Polly yesterday, when you had her in the hoist, how did you feel about how she was?”*

*Sybil: “Well she wasn’t comfortable. She was frightened, but I didn’t know how else we were actually going to get her off the bed and onto the chair. So, I think it’s a case of having to try and reassure people that they’re safe, and that they’re actually secure, and that they’re not going to fall out.”*

Although Sybil could not have made Polly happy with the use of the hoist, she recognised that by telling Polly what she was doing throughout the lift she could have made her more accepting and less frightened. The dilemma of having no immediately available alternative means of safely moving Polly prevented Sybil from meeting a number of Polly’s PNs, including: expressing emotions, acceptance, safety, and security.

The fourth type of ‘ducking’ occurred when patients’ PNs disrupted the nurse’s plans for the shift. When the nurses focussed on ‘*getting their work done’* rather than the patient’s individual needs, they failed to provide the patient with the care they required. This usually happened because nurses felt there was pressure on them that ‘*they must complete a set of duties during their shift’.* If a patient had an unexpected PN this gave the nurse an extra duty to manage which could disrupt their plans for the day. In order to prevent this disruption, nurses ignored patients’ PNs. This situation occurred when Julie (AuxN) had assisted Teresa with a shower and to return to her bedside, where the doctor then attended to her:

### Fieldnotes

*When the doctor left, I went behind the screens to put Teresa’s Lidocaine patch on. Teresa was very upset. I sat down in the chair beside Teresa’s bed and had a long chat with her. Teresa told me all about: her fears for the future, especially that she ‘wouldn’t be able to cope at home’; how difficult she’d found her illness; her family difficulties; and why she had such a lack of support. Teresa cried throughout this conversation and was visibly distressed.*

*During this conversation Julie came in and out three times to put things in Teresa’s locker, tidy things away, and leave the hairdryer.*

*At another point later in the conversation Rhona, the nurse in charge of the team this morning, shouted ‘Hazel, we’re away for tea, here’s the keys’. Her hand appeared under the curtains with the keys.*

*Later Julie said ‘I didn’t want to disturb you to say we were away for our tea, ‘cause I could see you were in something deep’.*

It is noteworthy that Julie found it acceptable to interrupt an in-depth conversation in order to tidy up after Teresa’s shower, but not to actually disturb the conversation, leaving the more senior nurse to do this. On other occasions disruptions like these, or a patient’s awareness that their PNs were holding the nurse back from her work, could inhibit patients from requesting psychosocial support.

The final reason the nurses gave for ‘ducking’ was paternalism: the nurse did not respond because they thought this would be too upsetting for a patient, or they felt they knew what was best for the patient. One example of this occurred during an episode of care with Vera, a patient who had been admitted to the ward for one week’s respite. She had deteriorated shortly after her admission, but was back to her normal state of health by the time of the multi-disciplinary team meeting (MDTM). The suggestion was made that Vera’s respite should be extended for both her and her husband’s sake, although she wished to return home on the originally planned day of discharge. However, the general consensus among the MDTM was that Vera’s admission should be prolonged.

### Fieldnotes

*Maria (RGN) told me ‘I’m going to have another chat with Vera and try to persuade her to stay in a bit longer. But I want to make sure we have plenty time to do this.’ Maria planned her morning’s care to allow time to spend with Vera to discuss her discharge date. Despite Maria’s attempts to negotiate that Vera should stay in the Hospice longer, Vera was very insistent and still said ‘I'd like to go on Friday’.*

Despite Vera’s clarity of choice the team decided it would be in her best interests to stay in the hospice longer and Vera’s choice was denied. The outcome of this was a frustrated and mistrusting patient, a husband who agreed with a foregone conclusion, and a nurse who had to obey the paternalism from the ward hierarchy and duck the patient’s PN.

In summary, the common factor in all of the ‘ducking’ encounters was that the nurses and patients did not share an acknowledgement that a PN existed.

### Discussion

The 4D categorisation demonstrates for the first time how PNs are responded to in practice. This study has demonstrated that patients’ PNs are rarely expressed to nurses as a standalone entity, which is how they are usually explained in nursing textbooks [51–53]. PNs arise during the various aspects of practice and are often subtly implied. This subtle expression of PNs contributes, at times, to the inability of nurses, in this study and others [31, 54], to recognise a request for psychosocial support. Conversely, nurses were observed providing holistic care by recognising and responding to patients’ PNs in a way that required much skill. The varied use of the 4Ds by individual nurses, even within one episode of care, indicates response does not depend upon nurses’ roles, education or belief that ‘it was their place to provide psychosocial support’. Both registered and auxiliary nurses gave the range of 4D responses, though AuxNs use of ‘deferring’ and ‘diverting’ responses were limited. Auxiliary nurses felt psychosocial support was part of their remit but felt they were ‘letting the patient’ down if they could not immediately support their needs. Educating AuxNs in the provision of psychosocial support within the reality of the organisational challenges of care could reduce their use of ‘ducking’ responses.

The study suggests that there may be some association between the type of PN expressed and the response given. For example, the most noticeable difference in the type of PN categories related to expression PNs which were least likely to be ‘dealt’ with immediately. Nurses’ hesitancy in dealing with difficult emotions verifies findings of previous studies into nurses’ palliative psychosocial support which found nurses lacking in confidence to deal with the difficult issues [37, 55], regardless of whether they had been educated in this area [56, 57]. One reason nurses attribute to lack of dealing with PNs, especially those relating to emotional expression, is ‘*not knowing a patient’* [58, 59]. However, despite this idea being repeated by the nurses - 37 of the 38 participating stated this claim – the idea that familiarity is required to provide psychosocial support was unproven [60].

The other challenges faced by the nurses in this study relates to balancing psychosocial support with the organisational demands of working as nurse in a ward. Examples have been included in this paper which demonstrate nurses facing the dilemma of following ward routines and completing their work for the day or meeting patients’ PNs.

### Limitations

This study is limited in that it only gives an overview of the PNs observed by one researcher, in one hospice ward, using convenience sampling. However, no claim is made that this is an exhaustive list of PNs, or that these findings are generalisable to other settings. The snapshot provided illustrates how PNs are expressed and responded to as part of ward nurses work. The challenges presented by the participant-observation methodology were minimised as much as possible. The potential of incorrectly recording observations was reduced by the collection of other data, especially matched interviews, carried out as soon as possible after the care, with the patients and nurses involved and about their interaction. Participant verification [61, 62] of the overall findings was carried out by feedback sessions to the nurses and observer impact reduced by the researcher’s experience and the time taken to develop the team’s ways of working.

## Conclusions

This study has allowed an exploration of the actual PNs of patients in a hospice setting and the way in which they were expressed. This paper also demonstrates how nurses respond to PNs. The participating nurses, who work in an area which has a key aim of providing psychosocial support, faced the challenge of responding to PNs whilst carrying out the other duties of their shift. The PNs were clearly associated with the palliative stage of the patients’ conditions. The idea that nurses can provide psychosocial support as an inherent component of practice was verified. The data included in this paper, and the discussions around the observed care, provides nurses everywhere with an example against which to compare their own practice.

## Abbreviations

AuxN: auxiliary nurses
HCP: healthcare professional
RGN: registered nurses
PN: psychosocial need
